# Novel Insights into the Role of Kras in Myeloid Differentiation: Engaging with Wnt/β-Catenin Signaling

**DOI:** 10.3390/cells12020322

**Published:** 2023-01-14

**Authors:** Noriko Yokoyama, Hitoshi Nakayama, Kazuhisa Iwabuchi

**Affiliations:** 1 Institute for Environmental and Gender Specific Medicine, Graduate School of Medicine, Juntendo University, Urayasu 279-0021, Chiba, Japan; 2 Infection Control Nursing, Graduate School of Health Care and Nursing, Juntendo University, Urayasu 279-0012, Chiba, Japan; 3 Laboratory of Biochemistry, Faculty of Health Care and Nursing, Juntendo University, Urayasu 279-0023, Chiba, Japan

**Keywords:** HL-60 cells differentiation, wild-type Kras, Wnt/β-catenin, tumor suppressor, hematopoietic stem cells, acute myeloid leukemia

## Abstract

Cells of the HL-60 myeloid leukemia cell line can be differentiated into neutrophil-like cells by treatment with dimethyl sulfoxide (DMSO). The molecular mechanisms involved in this differentiation process, however, remain unclear. This review focuses on the differentiation of HL-60 cells. Although the Ras proteins, a group of small GTP-binding proteins, are ubiquitously expressed and highly homologous, each has specific molecular functions. Kras was shown to be essential for normal mouse development, whereas Hras and Nras are not. Kras knockout mice develop profound hematopoietic defects, indicating that Kras is required for hematopoiesis in adults. The Wnt/β-catenin signaling pathway plays a crucial role in regulating the homeostasis of hematopoietic cells. The protein β-catenin is a key player in the Wnt/β-catenin signaling pathway. A great deal of evidence shows that the Wnt/β-catenin signaling pathway is deregulated in malignant tumors, including hematological malignancies. Wild-type Kras acts as a tumor suppressor during DMSO-induced differentiation of HL-60 cells. Upon DMSO treatment, Kras translocates to the plasma membrane, and its activity is enhanced. Inhibition of Kras attenuates CD11b expression. DMSO also elevates levels of GSK3β phosphorylation, resulting in the release of unphosphorylated β-catenin from the β-catenin destruction complex and its accumulation in the cytoplasm. The accumulated β-catenin subsequently translocates into the nucleus. Inhibition of Kras attenuates Lef/Tcf-sensitive transcription activity. Thus, upon treatment of HL-60 cells with DMSO, wild-type Kras reacts with the Wnt/β-catenin pathway, thereby regulating the granulocytic differentiation of HL-60 cells. Wild-type Kras and the Wnt/β-catenin signaling pathway are activated sequentially, increasing the levels of expression of C/EBPα, C/EBPε, and granulocyte colony-stimulating factor (G-CSF) receptor.

## 1. Introduction

HL-60 cells were originally derived from a patient with acute promyelocytic leukemia (French-American-British-classification M2, FAB-M2) [1]. These neutrophilic promyelocytes can be differentiated into several different cell types, including neutrophil-like, monocyte-like, and eosinophil-like cells depending on differentiation inducers. For example, HL-60 cells can be differentiated into neutrophil-like granulocytic cells upon exposure to polar compounds such as dimethyl sulfoxide (DMSO) and retinoic acid [2,3]. To date, however, the molecular mechanisms responsible for the myeloid differentiation of HL-60 cells remain unknown. Elucidation of this differentiation mechanism in acute myeloid leukemia (AML) cells is indispensable in the development of effective treatments. Upregulation of the p21 proteins encoded by *Ras* genes has been observed during the differentiation of HL-60 cells with DMSO, all-trans-retinoic acid (ATRA), and 12-O-tetradecanoylphorbol-13-acetate (TPA) [4]. 

Two members of the *Ras* gene family, *Nras* and *Kras* are frequently mutated in AML and related myelodysplastic syndromes [5]. The Ras proteins are small guanine nucleotide-binding proteins, with three ubiquitously expressed Ras isoforms, H-Ras, N-Ras, and K-Ras, identified to date, and oncogenic K-Ras being the most widely expressed. Mutations in *Ras* genes result in the expression of activated Ras proteins, which transduce signals for cell proliferation and cell survival [6,7]. Oncogenic mutants of *Kras* have been found to play essential roles during malignant transformation in human cancers [7,8,9]. In contrast, the knockout of *Ras* genes has shown that wild-type Kras (WT-Kras) is involved in normal mouse development, whereas Hras and Nras are redundant [10]. Importantly, Kras conditional knockout mice develop profound hematopoietic defects, including splenomegaly, an expanded neutrophil compartment, and a reduced number of B cells, indicating that WT-Kras is required for adult hematopoiesis [11]. Furthermore, hematopoietic cell-specific deletion of Kras was found to impair B cell development, but has no effect on T cell development [12], suggesting that WT-Kras plays distinctly different roles in hematopoietic stem cells [13,14,15]. However, the contributions of the WT-Kras signaling pathway to the differentiation processes in HL-60 cells have not been determined.

The Wnt signaling cascade is involved in many cellular events, including development, proliferation, differentiation, and migration [16,17,18,19]. Several reviews have described the roles of Wnt signaling in normal hematopoiesis and lymphocyte development [20,21,22], as well as the involvement of Wnt signaling in malignant tumors, such as leukemia and myeloma [18,23,24,25,26]. Increasing evidence indicates that the Wnt/β-catenin signaling pathway is deregulated in hematological malignancies. Wnt signaling in AML is deregulated at various levels, involving extracellular factors, cell surface receptors, cytoplasmic components, and activators of transcriptions [26,27,28]. Extracellular factors include ligands, agonists, and antagonists; cytoplasmic components include adaptors and components of destruction complexes involved in β-catenin stability and nuclear translocation. For example, elevated β-catenin levels have been observed in many patients with AML, although cells from these patients differ in sensitivity to β-catenin modulation. Thus, agents targeting β-catenin may not be successful in all patients with AML [29].

Our previous studies revealed that WT-Kras engages with Wnt/β-catenin signaling to regulate the DMSO-induced differentiation of the HL-60 human AML cell line [30]. At present, the molecular mechanisms that link these two pathways are not completely understood. The functions of both pathways are very delicately and finely regulated to control many cellular events. In this review, we are paying attention to the roles of tumor suppressors rather than tumor enhancers, although the functions of WT-Kras have not been fully elucidated. Ras proteins have been dismissed as an undruggable target for many decades. The great discovery of Kras G12C inhibitor [31] brings light to many patients with solid tumors, but much fewer benefits to patients with AML. Here, we have shown that WT-Kras is linked to the Wnt/β-catenin signaling pathway and regulated DMSO-induced differentiation of HL-60 cells. Engagement of WT-Kras and Wnt/β-catenin signaling may govern the downstream signaling pathway. We have described and discussed here the functions of tumor suppressors in these two signaling pathways, as well as our findings.

## 2. Distinguishing the Functions of Kras from Those of Other Members of the Ras Family 

The *Ras* genes are involved in many cellular processes, such as cell proliferation, differentiation, and apoptosis [7,32,33,34]. Oncogenic *Ras* mutations are the most common molecular alterations in human cancers. Wild-type Ras proteins have intrinsic GTPase activity, hydrolyzing bound guanine triphosphate (GTP) to guanine diphosphate (GDP) and thereby inactivating Ras growth-promoting signaling [6,34]. In contrast, mutant Ras is locked into the GTP-bound state, resulting in constitutive Ras signaling [7,33,34,35]. Thus, Ras proteins modulate downstream signal transduction by cycling between their GTP-bound active forms and GDP-bound inactive forms [6]. 

The three members of the *Ras* gene family, *Hras*, *Kras*, and *Nras*, are activated by mutations in many types of human cancers [7,34]. These three ubiquitously expressed Ras isoforms are highly homologous, although they have specific and distinct molecular functions [7,36]. Deletion of *Kras* is lethal for mouse embryogenesis, whereas *Hras* and *Nras* are not required for normal development [37]. *Hras* (-/-)/*Nras* (-/-) mice, expressing only *Kras* genes, were found to grow normally without any obvious mutant phenotype [10]. These results indicate that the functions of Kras differ from those of Hras and Nras, with WT-Nras and WT-Kras performing distinct, non-overlapping functions in cells [7,36]. Mutations of *Nras* and *Kras* are frequently observed in AML and related myelodysplastic syndromes [5]. Although these mutation patterns are different from those observed in solid tumors, the precise roles of *Ras* oncogenes in leukemogenesis remain unclear. The most well-established Ras effector pathways are the mitogen-activated protein kinase (MAPK) and phosphatidylinositol-3 kinase (PI3K) signaling pathways [34,38,39]. WT-Nras acts through Raf and RhoA and regulates adhesion, whereas WT-Kras coordinates motility through AKT and Cdc42 [36].

The 24 C-terminal amino acids of each Ras isoform contain information allowing them to target specific locations on cell membranes [40]. These hypervariable regions contain the C-terminus CAAX motif, which is modified by three steps: farnesylation, AAX proteolysis, and carboxyl methylation of the resulting C-terminal prenylcysteine [41]. These modifications convert the hydrophilic Ras protein to hydrophobic at their C-termini, allowing Ras proteins to associate with the membrane. Ras proteins must be anchored to the inner leaflet of the plasma membrane for full biological activity. 

Kras is expressed as two splice variants, Kras4A and Kras4B, with the latter being the more highly expressed isoform. Kras4A is highly expressed in many cell lines, especially in colon carcinoma and melanoma cell lines, despite *Kras4B* mRNA being more abundant overall [42]. In addition to the CAAX motif, the C-terminus of Kras4A contains a site for palmitoylation (C180), as well as bipartite polybasic regions (PB1, aa 167–170, and PB2, aa 182–185). Thus, WT-Kras4A has two membrane-targeting motifs [42]. WT-Kras4B localizes to the plasma membrane by forming a C-terminus farnesyl-cysteine carboxyl-methyl ester with a polybasic stretch of hexa-lysine residues (aa 175–180) [42,43,44]. Kras4A and Kras4B become oncogenic following their mutation. The polylysine domain of Kras4B is important for its transforming activity [42]. Kras4A is expressed in human leukemia cell lines and AML cells harboring Kras mutations [45]. Because the biological activity of Kras requires its localization to the plasma membrane [40,41,42], agents that interfere with this localization may have potential in the treatment of patients with AML [42,45]. Indeed, mutations at the palmitoylation site of oncogenic Kras4A (Kras4AG12D/C180S) significantly abrogate leukemogenesis, although these mutations still induce leukemia, albeit with a much longer latency [45]. Double mutations at the palmitoylation site (C180) and the KIKK motif (aa 182–185) abolish the oncogenic activities of Kras4A, indicating that the KIKK motif also contributes to the transforming activity of Kras4A [45]. Further analysis is required to understand the significant differences between these two variants [46]. Knowledge about their biochemical properties and biological functions may accelerate the development of more effective anti-leukemia treatments.

Oncogenic *Kras,* which mediates malignant transformation in human cancers [7,8,9], induces tumor cell migration by activating the MAPK and PI3K/AKT pathways [35,47]. Oncogenic Kras acts via cell-extrinsic mechanisms to enhance tumor progression by cross-talk with the tumor microenvironment [48], in addition to acting via cell-intrinsic mechanisms [49,50]. Colorectal cancer cells carrying oncogenic Kras trigger functional reprogramming of tumor-associated macrophages (TAMs), promote tumor progression, and induce the resistance of tumor cells to therapy [48]. In contrast, WT-Kras has tumor suppressor activity and is frequently lost during lung tumor progression [13]. WT-Kras inhibits oncogenic Kras-induced acute T-cell leukemia, indicating that WT-Kras can act as a tumor suppressor [14]. Loss of WT-Kras during oncogenic Kras-induced leukemogenesis facilitates the activation of all Ras isoforms [15]. Although WT-Kras was shown to act as a tumor suppressor in HL-60 cells, the functional significance of WT-Kras protein has not been fully determined [30].

## 3. Wnt Signaling Pathways

Three distinct Wnt-signaling pathways have been described to date: the Wnt/β-catenin signaling pathway, which is canonically mediated by β-catenin and either T-cell factor (Tcf) or lymphoid enhancer factor (Lef); the planar cell polarity (PCP) pathway; and the Wnt-Ca^2+^ pathway [17,18,21,51]. 

### 3.1. Wnt/β-Catenin Signaling Pathway

**Figure 1:**
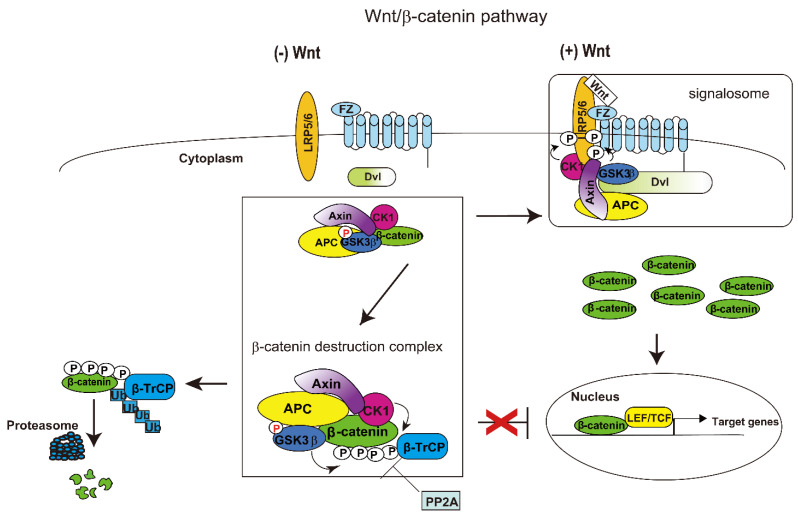
Wnt/β-catenin signaling pathway. The (**left side**) shows signaling in the absence of Wnt. In the absence of Wnt signals, β-catenin is degraded by a complex of proteins comprising APC, Axin, CK1, GSK3β, β-catenin, and β-TrCP (the E3-ubiquitin ligase). The rectangle shows the β-catenin destruction complex. Tyrosine phosphorylated GSK3β is active. Sequential phosphorylation of β-catenin by CK1 and GSK3β triggers its ubiquitination by the β-TrCP complex. Ubiquitylated and phosphorylated β-catenin is degraded by the proteasome. PP2A dephosphorylates β-catenin and inhibits its degradation. Red “P” shows tyrosine phosphorylation. The (**right side**) shows signaling in the presence of Wnt. Wnt ligand binds to its cognate receptors, FZ and Lrp5/6, forming a multiprotein complex known as the signalosome. Lrp5/6 receptors are phosphorylated by CK1α and GSK3β. The cytoplasmic protein Dishevelled (Dvl) is recruited to the plasma membrane and interacts with the FZ receptor and other Dvls. Interactions of Axin with phosphorylated Lrp5/6 and Dvl polymer inactivate the destruction complex. Consequently, β-catenin is stabilized and translocated into the nucleus. β-catenin associates with the Lef/Tcf transcription factor and activates the transcription of target genes. Black “P” shows serine/threonine phosphorylation. The model presented is a modified version and part of a previous model (FASEB Bioadvances, 2019; 00:1–15).

The Wnt/β-catenin signaling pathway is indispensable in embryonic development and homeostasis, including cell proliferation, differentiation, and apoptosis [16,17,18,19,52,53]. Control of this pathway is required to maintain normal hematopoiesis [20,21,22,54], with activation of Wnt/β-catenin signaling upregulating transcription factors and cell cycle regulators in normal hematopoietic stem cells (HSCs) [55,56]. The protein β-catenin is a key player in this Wnt cascade. The stability of β-catenin is regulated by a dynamic multiprotein assembly called the β-catenin destruction complex, which is composed of APC, β-catenin, GSK3β, Axin1, and casein kinase1α (CK1α) (Figure 1) [18,51,52,56,57]. 

In the absence of Wnt, free cytoplasmic β-catenin is rapidly targeted for degradation by the destruction complex. The protein phosphatase 2A is also associated with this complex. CK1 initially phosphorylates β-catenin at Ser45, priming the GSK3β catalyzed phosphorylation of β-catenin at Thr41, which in turn sequentially phosphorylates β-catenin at two other sites, Ser37 and Ser33. These sequential phosphorylations of β-catenin generate a recognition site for the E3-ubiquitin ligase β-TrCP, with ubiquitinated β-catenin subsequently degraded by proteasomes (Figure 1, left side) [58,59].

Once Wnts bind to their receptors, β-catenin destruction complexes are converted to receptor-associated Wnt signalosome complexes, resulting in the nuclear accumulation of β-catenin and the expression of Tcf/Lef target genes (Figure 1, right side) [60,61,62,63,64]. The binding of Wnt to its cell surface receptors, consisting of Frizzled (FZ) and LRP5/6, triggers the phosphorylation of PPPSPxS motifs in the cytoplasmic tail of LRP5/6 by GSK3β-primed CK1. These phosphorylation events provide a docking site for Axin. The protein Dishevelled (Dvl) is recruited to the plasma membrane and forms multiprotein complexes, called the Wnt signalosome, with FZ receptor, Axin, phosphorylated LRP5/6, and APC. The phosphorylation of β-catenin by GSK3β is suppressed, resulting in an accumulation of β-catenin in the cytoplasm [62]. This accumulated β-catenin translocates to the nucleus, where it regulates gene expression in cooperation with members of the Tcf and Lef family of transcription factors (Figure 1, right side) [18,62,65]. 

### 3.2. Non-Canonical Wnt Signaling Pathways 

**Figure 2:**
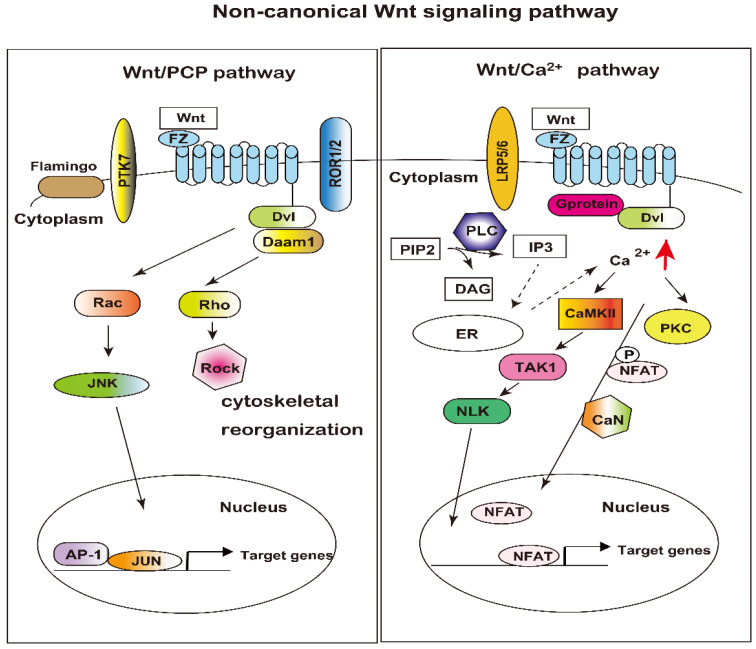
Non-canonical Wnt signaling pathways. **Left panel**: Wnt/PCP pathway; the binding of Wnt to FZ receptor activates the recruitment of Dvl and interacts with Daam1, thereby stimulating a cascade involving the small Rho GTPase family members Rho and Rac, as well as JNK. The PCP pathway regulates actin polymerization and cytoskeletal modifications. **Right panel**: Wnt/Ca^2+^ signaling pathway; upon Wnt binding to an FZ receptor, Dvl and G-protein are recruited to the receptor. PLC activation results in an elevation of IP3, which triggers Ca^2+^ release from the ER. Elevated Ca^2+^ level in the cytoplasm stimulates PKC, CaMKII, and Calcineurin, activating the transcriptional regulator NFAT. Elevated cytoplasmic Ca^2+^ also activates TAK1 and the Nemo-like kinase (NLK) signaling pathway through CaMKII. NLK phosphorylates Tcf/Lef, inhibiting the formation of the β-catenin-Tcf/Lef complex.

The hallmark of the non-canonical signaling pathways is their β-catenin-independent actions. In contrast, the canonical signaling pathways involve β-catenin (β-catenin-dependent). However, the difference between these two pathways is not clear-cut and rigorous, because some of the proteins linked to non-canonical Wnt signaling are also involved in canonical signaling. Two non-canonical pathways have been identified to date: the planar cell polarity pathway (Wnt/PCP) and the Wnt-calcium (Wnt/Ca^2+^) pathway (Figure 2). 

#### 3.2.1. Planar Cell Polarity (Wnt/PCP) Pathway

The Wnt/PCP pathway regulates the cytoskeleton, cellular shape, and migration. Non-canonical Wnt ligands, such as Wnt5a and Wnt11, bind to FZ receptors associated with various co-receptors, including pseudo tyrosine kinase7 (PTK7), receptor-like tyrosine kinase (RYK), receptor tyrosine kinase-like orphan receptor (ROR1/2), and Flamingo. These complexes associate with cytoplasmic adaptors, such as Dvl and Dvl-associated activator of morphogenesis 1 (Daam1). Signals are transduced Rac, Rho small GTPases, and Jun-N-terminal kinase (JNK), resulting in actin polymerization and cytoskeleton modification. Rho activates Rho-associated kinase (Rock), a major regulator of the cytoskeleton (Figure 2, left panel) [66]. Rho regulates cytoskeletal rearrangement and cell survival; in addition, Rac and subsequent JNK activation positively regulate AP-1-dependent gene transcription. 

#### 3.2.2. Wnt Calcium (Wnt/Ca^2+^) Pathway

This pathway accompanies an increase in the intracellular Ca^2+^ level upon binding of the Wnt ligand to the FZ receptor (Figure 2, right panel). Nuclear factor of activated T-cells (NFAT) and TGF-beta-activated kinase1 (TAK1)-induced Nemo-like kinase (NLK) are calcium-regulated transcription factors of the non-canonical pathway. Ligands binding to the receptor, Dvl, and G protein are recruited to the receptor and then activate phospholipase-C (PLC). PLC cleaves phosphatidylinositol 4, 5-bisphosphate (PIP2) into 1, 2-diacylglycerol (DAG) and inositol 1, 4, 5-triphosphate (IP3). In turn, IP3 causes the release of Ca^2+^ from ER, and IP3 and DAG together activate Protein kinase C (PKC), Ca^2+^/Calmodulin-dependent protein kinase II (CaMKII), and Calcineurin, and consequently activate the NFAT transcription factor. Activated genes via the Wnt/Ca^2+^ pathway regulate cell fate and cell migration [67].

## 4. Wnt/β-Catenin Signaling in Normal Hematopoiesis and AML

Wnt signaling plays an important role in normal hematopoiesis. The involvement of Wnt/β-catenin signaling in leukemia stem cell development remains unclear. The β-catenin/Tcf4 complex directly regulates the level of granulocyte colony stimulation factor (G-CSF) receptor and controls the process by which myeloid progenitor cells differentiate into granulocytes during both steady-state and emergency granulopoiesis [68]. Dominant negative Tcf4 (dnTcf4) abrogates the interaction of β-catenin with Tcf4, thereby reducing granulocytic differentiation. The dnTcf4 progenitors reduce the G-CSF receptor by downregulating the expression of the *Csf3* gene, which encodes the G-CSF receptor, as well as by attenuating STAT3 phosphorylation of the G-CSF receptor, thereby impairing the G-CSF receptor-mediated differentiation [68]. These findings provide novel insights into the regulation of granulopoiesis under steady-state and emergency conditions. CEBPα-deficient mice also show a lack of granulocyte differentiation and G-CSF signaling [69], with β-catenin/Tcf4 mediated transcription failing to compensate for the C/EBPα deficiency [70]. 

Aberrant Wnt signaling is involved in the pathogenesis of various cancers, including AML. The Notch, Wnt, and Hedgehog signaling pathways are particularly important in AML-associated stem cells [71]. Molecules associated with the Wnt/β-catenin signaling pathway, such as β-catenin, LEF-1, phosphorylated-GSK3β, and AXIN2, are considered prognostic markers of AML [26]. Because the expression of β-catenin depends on the type of AML, treatments targeting β-catenin are not always effective.

The protein β-catenin is at the core of the Wnt/β-catenin signaling pathway. Fold changes in β-catenin levels, rather than absolute levels, are critical for target gene activation [72,73]. Elegant studies with distinct mutants of *Apc* indicate that Wnt/β-catenin signaling regulates hematopoiesis in a dose-dependent manner [74]. Only a slight, ~2-fold increase in Wnt signaling is required to sustain normal HSC function, whereas intermediate, ~4-fold increases in Wnt signaling enhance myeloid differentiation and impair HSC self-renewal. Greater, ~22-fold increases in Wnt signaling levels favor early T-cell differentiation, whereas much greater, ~72-fold increases impair HSC self-renewal and differentiation [74]. Wnt activity in HSCs is reported to be very low [75,76,77], with only about one-fourth of normal activity being sufficient for normal function, suggesting that the complete absence of Wnt signaling is detrimental to the functions of HSCs [25]. Thus, previously observed discrepancies may be due to the different levels of activation of the Wnt signaling pathway [25]. The complexities of Wnt signaling in hematopoiesis derive from both the type and concentration of Wnt, the sources of cells, and interactions with other signaling pathways [25]. Determinations of optimal kinetics and fine adjustments of dose are crucial for prospective translational applications. Careful analyses are required to clarify the causes of conflicting results and determine the fine adjustments of the Wnt signaling pathway in leukemia.

Aberrant activation of Wnt signaling results in malignancy and blocks differentiation. Cross-talk between oncogenic Kras and Wnt/β-catenin signaling has been reported in colorectal cancers, with activity mediated through GSK3β or LRP6 [78,79]. Kras^Val12^, an oncogenic mutant of Kras, enhanced the stability of β-catenin via PI3K and GSK3β and the formation of nuclear β-catenin/Tcf4 complexes [78]. To date, however, WT-Kras has not been reported to interact with the Wnt/β-catenin cascade.

## 5. Non-Canonical Wnt Signaling Pathways in AML

Although the Wnt/β-catenin signaling pathway was found to be involved in AML, the involvement of non-canonical pathways in AML is much less frequent. Wnt5a, an almost purely non-canonical Wnt ligand, was found to suppress cyclin D1 expression and negatively regulates B cell proliferation, suggesting that Wnt5a suppresses hematopoietic malignancies [80]. Lethal-giant-larvae 1 and 2 (LLGL1/2) are involved in HSC polarity and AML biology. Decreased LLGL1 expression is associated with poor prognosis in AML patients [81]. In contrast, LLGL2 mutations are important for the transformation of severe congenital neutropenia to AML [82]. PTK7, which is expressed in ~70% of AML patients, is involved in cell migration and increases resistance to apoptosis [83]. PTK7-deficient mice show a decreased pool of HSCs due to aberrant proliferation and migration [84].

Cirmtuzumab, a monoclonal antibody targeting the Wnt receptor ROR1, has shown excellent results in models of chronic lymphocytic leukemia (CLL) and is now in phase I clinical trial (NCY02860676, completed, results submitted but not yet posted). Other trials testing Cirmtuzumab in combination with either the BTK inhibitor Ibrutinib (NCT03088878, Phase 1b/II), or the BCL2 inhibitor Venetoclax (NCT04501939, Phase II) are currently ongoing.

Treatment with 6-benzylthioinosine (6-BT) and ATRA induced HL-60 differentiation through activation of the non-canonical Wnt/Ca^2+^ signaling pathway involving the elevation of intracellular calcium level and phosphorylation of CaMKII and PKC. However, 6-BT regulates expression through both canonical and non-canonical Wnt signaling pathways, indicating that the 6-BT and ATRA-induced leukemic cell differentiation does not specifically involve the non-canonical pathway [85]. 

## 6. The Role of GSK3 in Hematopoietic Stem Cells

GSK3β is a negative regulator of the Wnt/β-catenin signaling pathway and regulates many other cellular processes [86], including hematopoiesis [87,88]. GSK3s are constitutively active in resting stages, with their activity being regulated by phosphorylation and dephosphorylation. Many upstream kinases, including AKT, A-kinase, C-kinase, p70ribosomal S6 kinase (p70S6K), and p90 ribosomal S6 kinase (p90RSK), were found to inhibit GSK3 by phosphorylating either Ser21 (GSK3α) or Ser9 (GSK3β) [86,89]. Close to 100 substrates, including transcription factors, have been reported [90,91]. The knockdown of both GSK3α and GSK3β resulted in aggressive AML [92], although GSK3α deletion alone had no effect on hematopoiesis, and deletion of GSK3β resulted in myelodysplastic syndromes. GSK3β and GSK3α contribute to AML development by affecting Wnt/AKT/mTOR signaling and metabolism, respectively. GSK3 interacts with the Wnt/β-catenin signaling pathway at multiple levels. GSK3 acts as a key suppressor of the Wnt/β-catenin signaling pathway by phosphorylation of β-catenin, resulting in its degradation by proteasomes. In contrast, GSK3 promotes the phosphorylation of Lrp5/6, resulting in the stabilization of β-catenin. Thus, GSK3 functions in a wide range of cellular processes and can act as either a tumor suppressor or a tumor enhancer [86,91,93].

The differentiation agent ATRA has been used to treat patients with acute promyelocytic leukemia (APL), a subtype of AML [94,95]. The combination of ATRA with GSK3 inhibitor significantly enhanced ATRA-mediated AML (non-APL leukemia) differentiation and growth inhibition. Retinoic acid receptor (PAR) is a target for GSK3, with GSK3 regulating the expression and transcription activity of PAR [96]. 1,25-Dihydroxyvitamin D3 (1,25 D) can induce the differentiation of leukemia cells, and inhibition of GSK3 can enhance the 1,25 D-induced differentiation of AML [97]. Thus, combinations of GSK3 inhibitors with differentiation agents such as ATRA and 1,25 D may have clinical potential, despite the necessity of determining an optimum dose of GSK3 inhibitor. The use of GSK3 inhibitor has several major advantages. Various AML cells to are sensitive to GSK3 inhibitors and its inhibition preferentially inhibits the growth of AML cells, but not inhibiting the growth of normal hematopoietic progenitor cells [93,98,99]. Combinations of GSK3 inhibitors with other agents are more effective than GSK3 inhibitors alone [100].

The finding that GSK3β regulates programmed death ligand-1 (PD-L1) suggests that GSK3β inhibition may be effective in immunotherapy against cancers [101]. PD-L1 is phosphorylated by GSK3β, with phosphorylated PD-L1 triggering proteasome degradation of PD-L1 by β-TrCP. In addition, glycosylation of PD-L1 protects from GSK3β-dependent phosphorylation. Thus, the immunosuppressive activity of PD-L1 is modulated by ubiquitination and N-glycosylation. Non-glycosylated PD-L1 forms a complex with GSK3β and β-TrCP and is subsequently degraded [101]. PD-L1 is expressed in AML, with high expression being associated with a poor survival rate [102]. The knockdown of PD-L1 inhibits cell proliferation and induces apoptosis and G2/M cell cycle arrest. Furthermore, PD-L1 knockdown attenuates the expression of P13K/AKT, whereas overexpression of PD-LI enhances their expression [102]. 

Taken together, these findings indicate that GSK3β inhibitors are positive regulators of the immune response against cancers, as well as negative regulators of the immune response in autoimmune conditions [103]. GSK3β, therefore, plays multifunctional roles in the immune tumor microenvironment [101,102,103,104], an unsurprising result due to the diversity of the GSK3β substrate. 

## 7. CCAAT Enhancer-Binding Proteins (C/EBPs)

The CCAAT enhancer-binding proteins (C/EBPs) are modular proteins, each containing a carboxy-terminal leucine zipper dimerization domain, a DNA binding domain, and an N-terminal activation domain [105,106,107]. Six members of the C/EBP family, C/EBPα, CEBPβ, C/EBPδ, C/EBPγ, C/EBPɛ, and CHOP (C/EBPζ), have been found to promote the expression of genes involved in various cellular responses, such as proliferation, growth, and differentiation [105,107]. C/EBPs are active in hematopoietic differentiation, including in myelopoiesis and granulopoiesis. Neutrophilic granulocytes have a short life, and granulopoiesis is tightly regulated. C/EBPs also regulate cytokine expression in neutrophils [108] and can have pro-oncogenic or tumor suppressor activity [107,109], depending on specific conditions, such as cell type, microenvironment, type of dimerization (homo- or hetero-dimerization), and interactions with various regulatory proteins [107]. 

### 7.1. C/EBPα

C/EBPα is highly expressed in myeloblastoma, and C/EBPα-deficient mice fail to undergo myeloid differentiation [69], suggesting that C/EBPα acts as a molecular differentiation switch during early hematopoiesis [110]. Neutrophilic differentiation depends on C/EBPα, and Lef-1 directly regulates the expression of C/EBPα during granulopoiesis [111]. C/EBPα also upregulates the G-CSF receptor promoter in myeloid cells [112,113]. Hematopoietic cell-specific Lyn substrate 1 (HCLS1 or HS1) is highly expressed in human myeloid cells and is involved in G-CSF-triggered myelopoiesis. HCLS1 interacts with Lef-1, and G-CSF stimulation leads to the phosphorylation of HCLS1 and its translocation into the nucleus with Lef-1 [114]. Expression of C/EBPα is specifically upregulated during granulocytic differentiation but is rapidly downregulated during monocytic differentiation.

### 7.2. C/EBPβ

C/EBPβ-deficient mice show defective macrophage function [115], as well as impaired responses to G-CSF and granulocyte/macrophage-colony stimulating factor (GM-CSF). In response to in vitro stimulation with IL-3 and /or GM-CSF, C/EBPα-deficient cells generate granulocytes [113,116], suggesting that IL-3 and GM-CSF promote granulopoiesis through a C/EBPα-independent pathway. This pathway was found to be significantly attenuated by inhibiting C/EBPβ [117]. C/EBPα and C/EBPβ share conserved C-terminus regions, which are involved in dimerization and DNA binding [118,119,120]. Therefore, C/EBPβ may dimerize with C/EBPα and bind to the same target promoters, including those of genes encoding the G-CSF receptor, myeloperoxidase, and neutrophil elastase [112,121,122]. C/EBPβ is required for emergency granulopoiesis, such as during infection and bleeding, as well as in cancers and hematological malignancies [117,123]. C/EBPα strongly inhibits the cell cycle, whereas C/EBPβ shows less inhibitory effect. Interestingly, neutrophil apoptosis is enhanced in C/EBPβ-deficient mice, suggesting that C/EBPβ is involved in suppressing neutrophil apoptosis [106].

### 7.3. C/EBPε

C/CEBPε is expressed only in hematopoietic tissues and participates in neutrophilic but not monocytic differentiation [124]. C/EBPε is involved in the terminal differentiation and functional maturation of granulocyte progenitor cells. Functional neutrophils and eosinophils are absent from C/EBPε-deficient mice, although these mice develop normally [125]. Neutrophils from these mice have impaired chemotactic and bactericidal activity [125]. C/EBPε is a critical regulator of terminal granulopoiesis, with its expression peaking in mature neutrophils and macrophages [126]. Treatment of HL-60 cells with DMSO for three days resulted in the upregulation of both the C/EBPδ and C/EBPε genes [127]. C/EBPε regulation is a rate-limiting step during G-CSF-regulated granulocyte differentiation [128].

## 8. G-CSF and G-CSF Receptor

Another critical regulator of granulopoiesis is G-CSF, a cytokine that regulates the differentiation and proliferation of myeloid cells [129,130]. The levels of G-CSF are normally very low in humans [131] but are markedly increased in response to infection, inflammations, and stress [131,132,133]. G-CSF directs granulocytic differentiation by changing the ratio of C/EBPα to PU.1, a member of the Ets family of DNA-binding proteins that regulates the fates of macrophages and neutrophils [134]. Stabilized β-catenin blocks monocyte–macrophage differentiation by inhibiting PU.1-controlled transcription of genes, including those encoding CEBPα, GFI1, and Egr2 [135]. G-CSF, which is highly expressed in AML and in gastric and colon cancers [136,137,138], promotes the differentiation of neutrophils and accelerates the maturation of metamyelocytes, resulting in a rapid and continuous increase in circulating neutrophils in blood [139,140]. 

G-CSF mediates signals by binding to a single homodimeric receptor, G-CSF receptor (G-CSFR). G-CSFR does not have intrinsic tyrosine kinase activity. Upon ligand binding, G-CSFR undergoes conformational changes, leading to the activation of downstream signaling pathways, including the JAK/STAT, PI3K/AKT, and MAPK/ERK pathways [140,141,142]. Three box regions (Boxes 1–3) are located at the C-terminus of G-CSFR. Boxes 1 and 2 are involved in proliferation, mainly via JAK/STAT signaling, whereas box 3 is important for the differentiation of myeloid progenitor cells, mainly via PI3K/AKT and MAPK/ERK signaling [143]. Four tyrosine residues on G-CSFR (704, 729, 744, and 764) are phosphorylated upon receptor dimerization and Jak activation, providing additional docking sites for signaling [144,145]. Reviews have described each of these signaling pathways [140,141,142,146]. 

G-CSF has been found to regulate myeloid differentiation via C/EBPε [128]. G-CSFR has binding sites for C/EBPα and PU.1, regulating several myeloid gene promoters [112]. During emergency granulopoiesis, the levels of G-CSF are increased. G-CSFR signaling via JAK results in the translocation of phosphorylated STAT3 to the nucleus. Phosphorylated STAT3, in turn, directly stimulates the expression of Myc and C/EBPβ. C/EBPβ, in turn, stimulates Myc transcription and replaces C/EBPα at the Myc promoter, thereby inhibiting the C/EBPβ-induced transcriptional repression of Myc expression, enhancing myeloid progenitor cell proliferation and neutrophil generation [123,147]. Both G-CSF-dependent and -independent pathways regulate neutrophil maturation, as mice lacking both G-CSF and G-CSFR produce morphologically mature neutrophils, although at lower-than-normal levels [148]. Expression of other C/EBPs in these mice can compensate for the lack of a G-CSFR-mediated pathway. C/EBPs are required for granulopoiesis independent of the induction of G-CSFR [149]. Cytokine stimulation downregulates the expression of all members of the C/EBP family, except for C/EBPβ. Cytokine or infection-induced enhancement of granulopoiesis is impaired in C/EBPβ knockout mice [123]. C/EBPα activation of the C/EBPε gene may be unnecessary, as C/EBPα may be replaced by C/EBPβ under stress conditions [123,149]. 

## 9. WT-Kras Engages with Wnt/β-Catenin Signaling Pathway in the DMSO-Induced Differentiation of HL-60 Cells

DMSO-induced differentiation of HL-60 cells has been regarded as a model for the differentiation of myeloid cell lines. Because DMSO can affect the fluidity of plasma membranes, it can activate several biological functions of cells by removing surface water from phospholipid bilayers [150,151]. AMP-dependent and phospholipid/Ca^2+^-dependent protein kinases are thought to be involved in the early stages of regulation of the phosphorylation/dephosphorylation status in cells [152,153]. Surprisingly, however, little is known about the detailed mechanisms regulating the myeloid differentiation of HL-60 cells. Various signaling molecules are upregulated during the DMSO-induced differentiation of HL-60 cells in a time-dependent manner (Table 1). 

The amount of WT-Kras on the membrane and Kras activity is significantly enhanced after DMSO treatment for 1 day and is sustained for up to 5 days. These results indicate that the activation of Kras is the first event during the DMSO-induced differentiation of HL-60 cells. WT-Kras levels on the plasma membrane correlate with Kras activity (Table 1). GSK3β plays a pivotal role in the Wnt canonical pathway, with the phosphorylation of GSK3β at Ser 9 inhibiting its activity [17]. The phosphorylation ratio (p-GSK3β/GSK3β) at Ser 9 is enhanced by treatment with DMSO for 3~5 days (Table 1), suggesting that the Wnt/β-catenin pathway participates in the DMSO-induced differentiation of HL-60 cells. After the activation of Kras, significant inactivation of GSK3β was observed after 3 days of treatment with DMSO (Table 1).

Consistent with findings showing that β-catenin levels are low in HL-60 cells [154], basal levels of nuclear β-catenin were shown to be quite low, increasing after 1 day of treatment with DMSO and continuing to increase for 5 days (Table 1). Similarly, an increase in Tcf4 was observed during the differentiation of HL-60 cells. Accumulation of these molecules in the nucleus showed that the Wnt/β-catenin signaling pathway was activated, since cytoplasmic Wnt regulators, such as β-catenin, APC, and Axin, appeared transiently in the nucleus [155]. Wnt stimulation triggers the shuttling of Axin and β-catenin to the nucleus [63]. 

CEBPs are active during hematopoietic differentiation. Upregulation of C/EBPα was initially observed after β-catenin accumulation in the nucleus, with the levels of C/EBPε upregulated following the elevation of C/EBPα (Table 1). The expressions of C/EBPα and C/EBPε were found to overlap after 3 days of treatment with DMSO. These time lags in the expression of C/EBPα and C/EBPε have been reported previously [125,126]. Increased expression of G-CSFR protein was observed after 1 day of treatment with DMSO, with its expression subsequently increasing in a time-dependent manner (Table 1). Thus C/EBPα, C/EBPε, and G-CSFR are increased in tandem to stimulate granulocytic maturation during the differentiation of HL-60 cells.

## 10. Level of β-Catenin Regulates DMSO-Induced Differentiation in HL-60 Cells

GSK3β phosphorylation and CD11b expression are attenuated by treatment with Kras siRNA or Kras inhibitor [30]. Furthermore, the GSK3β inhibitor CHIR-99021 [156] was found to enhance CD11b expression in HL-60 cells differentiated into neutrophilic lineage (DHL-60 cell), presumably by the accumulation of stable β-catenin in the nucleus [30]. Both the PI3K inhibitor LY294002 and the AKT inhibitor Akti-1/2 attenuated CD11b expression, correlating with a reduction in the p-GSK3β/GSK3β ratio [30]. These findings indicate that PI3K/AKT is a downstream effector of Kras signaling and that AKT phosphorylates GSK3β [38,157,158]. The accumulation of β-catenin in the nucleus is an indicator of activation of the Wnt cascade [51,52,62]. Pyrvinium pamoate blocked the transcription of the β-catenin gene, whereas PKF115-584 inhibited β-catenin/Tcf4 interactions [159]. The expression of CD11b was reduced by either pyrvinium pamoate or PKF115-584, indicating that β-catenin is important in the differentiation of HL-60 cells [30]. Nuclear accumulation of β-catenin correlates with the degree of differentiation of HL-60 cells. Canonical Wnt signaling is mediated by the interaction of β-catenin with Lef/Tcf transcription factors and subsequent transcriptional activation of Wnt-target genes. Lef/Tcf-sensitive transcription activity is enhanced in DHL-60 cells, with this activity correlating with the accumulation of β-catenin in the nucleus (Table 1). Furthermore, treatment with Kras siRNA attenuated Lef/Tcf sensitive transcription activity, as well as CD11b expression [30]. Treatment with the Kras inhibitor SAH-SOS1 also attenuates CD 11b expression, indicating that Kras plays a positive role in the DMSO-induced differentiation of HL-60 cells [30]. Kras (G12C) inhibitor 12, which irreversibly binds to the oncogenic mutant Kras (G12C), blocks the interaction of the latter with Raf. This inhibitor does not affect WT-Kras, whereas its activity on mutant Kras depends on its binding to the mutant cysteine residue [31]. Preliminary data have shown that Kras (G12C) inhibitor 12 enhanced the expression of CD11b in a dose-dependent manner. Taken together, these data support a model by which the DMSO-induced differentiation of HL-60 cells is regulated by sequential activation of the WT-Kras and Wnt/β-catenin signaling pathways.

Increased levels of p-GSK3β stabilize β-catenin, which in turn acts as a nuclear transcriptional co-activator. Interactions with β-catenin and members of the Lef/Tcf family stimulated the transcription of various target genes, such as those encoding C/EBPα, C/EBPε, and G-CSFR, in DHL-60 cells. The basal level of β-catenin was found to be much lower in HL-60 cells than in other cancer cell lines [154], with levels in HL-60 cells increased 3~5 fold upon treatment with DMSO. Lower (~2.5-fold) activation of Lef/Tcf-sensitive transcription was observed in DHL-60 cells (Table 1). In agreement with previous findings [72,73,74], intermediate increases (3~5-fold) in the level of β-catenin triggered the DMSO-induced differentiation of HL-60 cells, despite the absolute concentrations of β-catenin not being sufficiently high. Fold increases in β-catenin levels were found to govern downstream signaling events, such as transcription activity and gene expression. Constitutively high levels of β-catenin produced by Wnt may result in super proliferation and tumorigenesis. These findings support the hypothesis that Wnt signaling dose and downstream signaling govern outcomes in myeloid cells [72,73,74,160]. Oncogenic Kras has frequently shown enhanced signaling through the Wnt pathway and mediates tumor development. WT-Kras may act to finely adjust β-catenin levels during HL-60 cell differentiation, indicating that WT-Kras regulates the DMSO-induced differentiation of HL-60 cells through the Wnt canonical pathway.

β-Catenin is also involved in apoptosis, as it acts as a molecular hub for multiple signals and regulates various cell functions. The proapoptotic activity of β-catenin has not been fully determined in hematopoietic cells [53]. Interestingly, activated β-catenin has been found to enhance apoptosis in HSCs [161], and the conditional expression of an active form of β-catenin was shown to enhance mitochondria-mediated apoptosis in HSCs/HPCs [162]. A Wnt agonist was found to induce apoptosis and cell death in the murine leukemia macrophage-like cell line RAW264.7 via the accumulation of β-catenin in the nucleus, although this agonist did not inhibit GSK3β activity [163]. In contrast, silencing of β-catenin induced autophagy and apoptosis in multiple myeloma cells, accompanied by increased expression of p53, active caspase3, and Bcl-2-associated X protein [164]. Different Wnt/β-catenin signaling dosages were found to regulate the hematopoiesis of HSCs, myeloid precursors, and T lymphoid precursors [74]. Thus, outcomes depend on dosages and types of reagents and cross-talk between the Wnt/β-catenin and other signaling pathways [25,73,74]. The mechanism by which β-catenin switches from differentiation to apoptosis of HL-60 cells is still unclear. Detailed analyses, including fine adjustments of Wnt/β-catenin doses, are required.

## 11. Applications to the Treatment of Patients with AML

The *Ras* genes are frequently mutated in cancers, with mutations in *Kras* being associated with the three most lethal types of cancer: lung, colorectal, and pancreatic cancers. Studies have sought to identify effective inhibitors of Ras, with these genes long thought to be “undruggable” targets. Most previous studies have focused on pancreatic and lung cancers, with direct inhibition of Ras considered a desirable approach for the treatment of tumors induced by mutant *Ras* genes. The identification of a novel allosteric binding pocket beneath the switch-II region in the Kras G12C protein [31] enabled the design of agents that could interact strongly to inhibit Kras activity. AMG510, the first inhibitor targeting Kras (G12C) protein to be tested in clinical trials, was approved by the U.S. Food and Drug Administration in 2021 to treat patients with nonsmall-cell lung cancer (NSCLC). This agent, along with several other specific Kras G12C inhibitors, such as MRTX849, ARS-3248, and LY3499446, are also undergoing clinical trials in patients with solid tumors, both as a single agent and in combination with other therapies [165,166]. In contrast, drugs directly targeting Ras are not yet undergoing clinical trials in patients with AML. Although to date, there are no effective therapies targeting Kras mutations in AML, our preliminary results showed that Kras G12C inhibitors enhanced DMSO-induced differentiation of HL-60 cells, suggesting that these inhibitors, possibly in combination with other agents such as palmitoylation, farnesyltransferase, and GSK3 inhibitors, may be effective. Differentiation of APL, a subtype of AML, was found to be induced by treatment with ATRA, with or without ATO [94,95], suggesting that treatments that enhance terminal differentiation and trigger apoptosis of leukemia cells may provide clues for the development of novel agents. Potential agents targeting protein palmitoylations, including Kras4A, are listed in Table 2.

Oncogenic Kras 4A possesses leukemogenic activity, and palmitoylation regulates its activity [45]. The palmitoylation inhibitor, 2-bromopalmitate (2-BP), may be effective, as double mutations at the palmitoylation site (C180) and KIKK motif (aa 182–185) of Kras4A were found to abolish oncogenic activity [45]. The FLT3 receptor tyrosine kinase and its ligand are important for the expansion of hematopoietic progenitor cells and the generation of mature natural killer cells and dendritic cells. Recently, FLT3 has become considered an important marker in AML. The binding of FLT3 ligands to an EC domain triggers dimerization and promotes the phosphorylation of the kinase domain (KD), thereby activating the receptor and several downstream signaling pathways, such as the PI3K/AKT and Ras/Raf/MEK/ERK cascades [170]. FLT3 has two functional domains, the juxtamembrane (JM) domain and the KD, with its most common mutation being an internal tandem duplication (ITD). The FLT3 inhibitor Gilteritinib (ASP2215) has been approved by the U.S. Food and Drug Administration for the treatment of patients with relapsed or refractory FLT3-mutated AML [171]. One drawback to the use of Gilteritinib is acquired drug resistance, resulting from the reactivation of the Ras/Raf/MEK/ERK pathway or a secondary mutation of Ras. Agents that target both FLT3 and Ras in AML are therefore needed. The depalmitoylation inhibitor, Palmostatin B, has been found to reduce cell surface expression, signaling, and cell growth associated with FLT3-ITD [169], making palmitoylation a potential therapeutic target [172]. Stimulation of differentiation triggered by activation of WT-Kras may be effective in the treatment of AML, although more detailed analysis is required. The development of more effective combination therapies requires characterizations of the molecules that act upstream or downstream of Kras signaling pathways in AML. 

Dysregulation of the Wnt/β-catenin signaling pathway has been observed in various malignancies, including AML. Clinical trials of agents targeting the Wnt/β-catenin signaling pathway in AML are shown in Table 3. Currently, there are no FDA-approved Wnt-targeting agents. Clinical trials of PRI-724, a CBP/β-catenin inhibitor, and CWP232291, an inhibitor of β-catenin transcriptional activity, have been completed, but their results have not yet been published. An additional clinical trial of CWP232291, both alone and in combinations with Lenalidomide and Dexamethasone, is currently underway in patients with AML and multiple myeloma (MM) (NCT02426723, Phase I), with preliminary results showing encouraging clinical activity [173]. The clinical trial of CWP232291 combined with the S-phase-specific anti-metabolite drug cytarabine is also under investigation. GSK3 inhibitors LY2090314 and ATRA combined with LiCl are currently underway. Treatment with GSK3 inhibitors reduced cell proliferation, enhanced apoptosis, and lowered drug resistance [98,99,174]. Other agents are still under investigation. 

FLT3, an upstream molecule of Ras, is also associated with the Wnt signaling pathway in myeloid transformations. Mutations in the *FLT3* gene have been detected in approximately 20~30% of patients with AML. FLT3 inhibitors can inhibit the phosphorylation of FLT3 and induce apoptosis [175]. FLT3-ITD mutations resulted in high levels of β-catenin protein and induced TCF-dependent transcription, indicating that FLT3-ITD synergizes with the Wnt-dependent pathway in myeloid transformation [176]. S-palmitoylated FLT3-ITD has been observed in AML cells, with palmitoylation of FLT3-ITD found to promote leukemia growth [169]. Cyclooxygenase-2 (Cox-2) is a key enzyme in prostaglandin production that has been implicated in tumorigenesis. Cox-2 stimulates Wnt signaling through prostaglandin E. A current clinical trial is testing the combination of Celecoxib and Doxorubicin (NCT03878524, Phase I) [177].

Although mutations in Wnt signaling components, such as APC, Axin, or β-catenin, are common in solid cancers such as colorectal, breast, and liver cancers, these mutations have rarely been reported in patients with AML and other hematological malignancies [160]. In AML, *β-catenin* mRNA is not deregulated, and its level does not correlate well with the level of β-catenin protein [178,179,180]. Because β-catenin protein levels in AML depend on post-translational mechanisms, agents targeting Wnt/β-catenin may be less effective in AML than in solid tumors. Combination therapy may be more suitable in AML, but a more detailed analysis of mechanisms regulating β-catenin expression in AML is needed. 

**Table 3:** Clinical trials involving Wnt signaling inhibitory/modulating compounds in AML.

Affected Target	Agent	Mechanism	Diseases	Clinical	References
*CBP/β-catenin*	PRI-724	Inhibits interactions between CBP and β-catenin and prevents transcription of Wnt target genes	AML, CML	NCT01606579 (Phase I/II) completed, no results posted	[181] [182]
*β-catenin*	CWP232291	Inhibits β-catenin transcriptional activity, leading to degradation of β-catenin and induction of apoptosis in leukemia cells	AML, CML	NCT01398462 (Phase I) completed, no results posted	[173]
*TBL1/β-catenin*	BC2059 (Tegavivint)	Inhibits β-catenin/transducin β-like 1 (TBL1) complex, degrades β-catenin and abrogates Wnt target gene expression	Refractory leukemia (AML)	NCT04874480 (Phase I) recruiting participants	[183] [184]
*β-catenin* *S-phase-specific anti-metabolite drug*	CWP232291+ AraC/cytarabine	Inhibit β-catenin transcriptional activity, induce apoptosis in leukemia cells	AML	NCT03055286 (Phase I/II) no results posted	
*GSK3*	LY2090314	Inhibits GSK3β, results consistent with Phase I trial results. Well tolerated, but no patients achieved CR or PR	AML	NCT01214603 (Phase II)	[100]
*PAR receptor* *GSK3*	ATRA+LiCl	Inhibit phosphorylation of PU.1, enhance leukemia cells differentiation	AML, APL	NCT01820624 (Phase I)	[185]
*FLT3*	Gilteritinib (ASP2215)	Inhibits FLT3 and AXL receptor, and inhibits cell growth	AML, relapsed or refractory FLT3-mutated AML	NCT03070093 (Phase I/II) completed (May 2021), approved for marketing NCT02421939, NCT03182244 (Phase III)	[171]
*Cyclooxygenase-2*	Celecoxib +doxorubicin	Inhibit cell growth and induce apoptosis in leukemia cell lines	AML, primary AML blasts	NCT03878524 (Phase Ib)	[177]

CR: complete remission; PR: partial remission. Amore detailed list of clinical trials can be obtained at www.ClinicalTrials.gov (accessed on 30 November 2022).

## 12. Perspective

**Figure 3:**
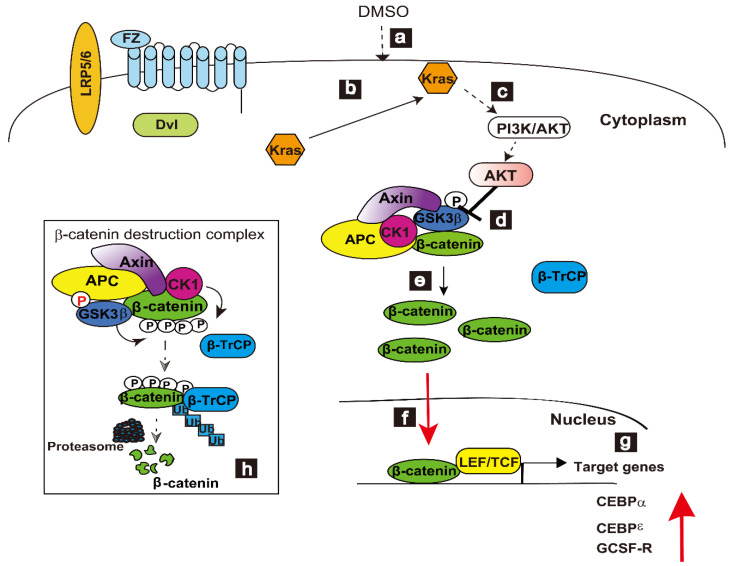
Association of WT-Kras with Wnt/β-catenin signaling networks in the DMSO-induced differentiation of HL-60 cells. DMSO triggers Kras activation through the recruitment of Kras to the plasma membrane (a,b). AKT phosphorylates GSK3β and inactivates GSK3β (c,d). Accumulated unphosphorylated β-catenin in the cytoplasm translocates to the nucleus (e,f), where it binds to Lef/Tcf and stimulates the transcription of target genes, such as those encoding CEBPα, CEBPɛ, and G-CSF receptor (g). In the absence of DMSO, β-catenin is phosphorylated by a destruction complex consisting of core proteins, including Axin, APC, CK1, β-catenin, GSK3β, and E3-ubiquitin ligase β-TrCP. Degradation of phosphorylated β-catenin follows its ubiquitination by proteasomes (h). Black “P” shows serine/threonine phosphorylation. Red “P” shows tyrosine phosphorylation. The model presented slightly modified a previous model (FASEB Bioadvances, 2019; 00:1-15).

The proposed pathways of DMSO-induced differentiation of neutrophilic HL-60 cells are schematically described in Figure 3 [30]. The main difference between the Wnt/β-catenin signaling pathway (Figure 1) and the DMSO-induced differentiation pathway was that the signal in the latter did not go through LRP5/6 and FZ receptors. Our model shows that the Kras signal triggers the inhibition of GSK3β via the PI3K/AKT pathway, indicating that Kras regulates DMSO-induced differentiation via the Wnt/β-catenin signaling pathway. 

Upon DMSO treatment, WT-Kras is recruited to the plasma membrane (Figure 3, (a,b)), enhancing Kras activity.

For full biological activity, WT-Kras proteins must be anchored to the inner leaflet of the plasma membrane. At present, the mechanism by which DMSO stimulates Kras translocation to the plasma membrane has not been determined. The stability of β-catenin is regulated by the β-catenin destruction complex (Figure 3, (d,h)). 

The WT-Kras signal is transduced by PI3K/AKT signaling, downstream of WT-Kras. AKT is a GSK3β kinase [157], with AKT phosphorylation of GSK3β resulting in the inactivation of GSK3β (Figure 3, (c,d)). The accumulated non-phosphorylated β-catenin (Figure 3, (e)) in the cytoplasm is translocated into the nucleus of DHL-60 cells (Figure 3, (f)). When GSK3β is phosphorylated by AKT, WT-Kras signaling is linked to the Wnt/β-catenin signaling pathway. Accumulated β-catenin interacts with Tcf/Lef, enhancing the expressions of the G-CSF receptors C/EBPα and C/EBPε, which are necessary to differentiate HL-60 cells. G-CSF receptor expression is regulated by β-catenin and the TCF4 complex [68]. Lef-1 enhances C/EBPα expression, and C/EBPα upregulates both the G-CSF receptor promoter and C/EBPε expression [111,112,113]. Thus, the transcription factors C/EBPα, C/EBPε, and G-CSFR are upregulated and involved in the granulopoiesis of HL-60 cells (Figure 3, (g)). C/EBPα expression is enhanced soon after treatment with DMSO, and its activity is sustained until the late stages of differentiation. Sustained C/EBPα expression contributes to the differentiation of bipotential myeloid precursors to granulocytes, not monocytes. C/EBPε, acting downstream of C/EBPα, is upregulated following the elevation of G-CSFR in DHL-60 cells. C/EBPα has been shown to upregulate the expression of both G-CSFR and C/EBPε [112,113]. The expression of C/EBPα partially overlaps that of C/EBPε to regulate the fine stage of differentiation [125]. C/EBPα also plays an important role in the expression of G-CSFR [69]. HL-60 cells differentiate into monocytes when C/EBPα mRNA levels are reduced [110]. 

In the absence of DMSO, phosphorylated β-catenin in HL-60 cells is degraded by a destruction complex of proteins, including GSK3β, APC, CK1, protein phosphatase 2A, and the E3-ubiquitin ligase β-TrCP (Figure 3, (h)). Thus, levels of β-catenin regulate the differentiation of HL-60 cells.

The expression of CD11b was shown to be attenuated by either Kras knockdown or a Kras inhibitor, as well as by PI3K/AKT inhibitors and small molecule inhibitors of β-catenin. In contrast, CD11b expression was enhanced by the GSK3β inhibitor. Thus, elevated β-catenin positively regulates the differentiation of HL-60 cells, with the level of β-catenin correlating with the differentiation status of DHL-60 cells. Moreover, Lef/Tcf-sensitive transcription activity is inhibited by treatment with siRNA against Kras, indicating that WT-Kras is linked to the Wnt/β-catenin signaling pathway. Thus, WT-Kras can enhance the stability of β-catenin, thereby increasing the nuclear level of β-catenin/Tcf4 complexes. 

In this review, we describe the pathway in which WT-Kras engages with the Wnt/β-catenin signaling pathway to enhance the differentiation of HL-60 cells by DMSO treatment. However, the differentiation of hematopoietic cells is regulated by many signaling pathways. The pathway we have presented here is one of the mechanisms. More detailed analysis is required to fully understand the mechanisms of HL-60 cell differentiation since HL-60 cells can be differentiated into several different cell types depending on inducers.

## 13. Conclusions

The Wnt/β-catenin signaling pathway is an evolutionarily conserved signal transduction cascade involved in normal human development and diseases. Additional studies are required to fully understand the roles of β-catenin in the hematopoiesis of HSC and myeloid lineage cells. Hyper-activation of Wnt signaling by mutations in genes encoding components of the β-catenin destruction complex, such as APC, Axin, and β-catenin, contributes to tumorigenesis. Mutations in these molecules in solid tumors resulted in the constitutive activation of this pathway. In contrast, such mutations are rarely reported in AML and other hematological malignancies [160]. However, β-catenin, the central effector of the Wnt signaling cascade, is highly involved in AML. AML is a markedly heterogeneous disease, and its classification is important in determining suitable therapy for individual patients. In addition to genetic heterogeneity, AML is associated with the deregulation of various signaling pathways. Elevated β-catenin activity via tyrosine phosphorylation by FLT3, β-catenin stabilization by γ-catenin, and high levels of LEF-1 promoting the nuclear localization of β-catenin are some examples of post-transcriptional mechanisms occurring in AML [160]. Moreover, spatial and temporal control of Wnt signaling is crucial to ensure correct outcomes during normal development and AML tumorigenesis. Future studies should focus on the identification of additional mutations in signaling molecules and the determination of the connections of various genotypes with specific signaling pathways. 

Following the discovery of Kras G12C inhibitors, Ras becomes a druggable target. Although Kras G12C inhibitors showed great success in solid tumors, therapies targeting Kras in AML are still under development. The fact that activation of WT-Kras can enhance the differentiation of AML cells may give some hope to patients with AML. Agents promoting terminal differentiation have markedly enhanced cure rates in patients with certain AML subtypes, such as ATRA treatment of patients with APL [94,95]. ATRA plus a GSK3 inhibitor significantly enhanced ATRA-mediated AML (non-APL leukemia) differentiation and growth inhibition [96]. 1,25 D plus GSK3 inhibitor also enhanced AML differentiation [97], suggesting that combination treatment may be more effective in AML. The identification of new therapeutic targets and drugs has led to many clinical trials of differentiation therapy in AML, such as inhibitors of mutant isocitrate dehydrogenases (IDH1/2) and FLT3 [186]. The involvement of WT-Kras in the Wnt/β-catenin signaling pathway during the differentiation of HL-60 cells suggests that combined terminal differentiation therapy may be effective for patients with AML. Possible candidates can include palmitoyl transferase inhibitors, GSK3 inhibitors, ATRA, and FLH3 inhibitors. Basic research focusing on fundamental phenomena, such as differentiation or apoptosis, can contribute to the development of novel therapy. Moreover, a fundamental analysis of AML is necessary for further advances in its treatment. 

## Figures and Tables

**Table 1:** Changes in various parameters in response to DMSO treatment in HL-60 cells.

DMSO treatment	0 day	1 day	3 days	5 days
Kras translocation (plasma membrane)	1.0	1.5	1.5	1.5
Kras activity (plasma membrane)	1.0	1.7	2.0	2.0
pGSK3β/GSK3β ratio at Ser9 (cytoplasm)	1.0	1.3	2.0	11.9
GSK3β/actin (cytoplasm)	1.0	1.0	1.0	1.0
β-catenin (nucleus)	1.0	3.2	4.3	5.6
TCF4 (nucleus)	1.0	1.3	1.8	4.6
C/EBPα (nucleus)	1.0	1.4	2.1	2.1
C/EBPε (nucleus)	1.0	1.1	2.5	2.1
G-CSFR (nucleus)	1.0	1.9	2.8	3.5
Lef/Tcf-sensitive transcription	1.0	N. D	N. D	2.5

For the activity of Kras, active forms of Kras were captured by Raf1 RBD agarose, and bound proteins were analyzed with an anti-Kras antibody. Protein expression was analyzed by Western blotting with antibodies to indicate various parameters. The ratio of protein expressions in HL-60 cell differentiated into neutrophilic lineage (DHL-60 cell) relative to HL-60 cell at t = 0 was shown. The results shown represent 4~6 independent experiments. N. D: not determined. For Lef/Tcf-sensitive transcription, HL-60 cells were cultured in a medium without or with DMSO for 4 days, and then transfected with Super8x TOPFlash (M50) for 1 day. Lef/Tcf-sensitive transcription activity was analyzed in cell lysates. The ratio of the activation of Lef/Tcf-sensitive transcription in DHL-60 to HL-60 cells is shown. Data shown here were modified based on the results in FASEB Bioadvances, 2019; 00:1-15.

**Table 2:** Potential therapies targeting palmitoylation in AML.

Target Protein	Agents	Mechanism	Diseases	References
Kras4A	2-bromopalmitate (2-BP)	Inhibits translocation to the membrane	AML, CML	[45]
LAT2	ABHD17A/B/C (ABD957, depalmitoylase inhibitor)	Palmitoylation of LAT2 increases leukemia cell proliferation	AML, APL	[167] [168]
FLT3-ITD	Gilteritinib+Palmostatin B (palm B, depalmitoylase inhibitor)	Increased palmitoylated FLT3-ITD inhibits leukemia cell growth	AML, primary AML blasts	[169]

## Data Availability

Not applicable.
